# *Punica granatum* L. Modulates Antioxidant Activity in Vitrified Bovine Ovarian Tissue

**DOI:** 10.3390/ijms27020903

**Published:** 2026-01-16

**Authors:** Solano Dantas Martins, Maria Alice Felipe Oliveira, Venância Antônia Nunes Azevedo, Francisco das Chagas Costa, Ingrid Gracielle Martins da Silva, Selene Maia de Morais, Sônia Nair Báo, José Roberto Viana Silva, Vânia Marilande Ceccatto, Valdevane Rocha Araújo

**Affiliations:** 1 Graduate Program in Biotechnology (PPGB), Federal University of Ceará, 100, Maurocélio Rocha Ponte Avenue, Sobral 62041-040, CE, Brazil; 2 Laboratory of Research in Reproductive Physiology (FisioRep Lab), Parnaíba Delta Federal University, 2819, São Sebastião Avenue, Parnaíba 64202-020, PI, Brazil; 3 Laboratory of Biotechnology and Physiology of Reproduction (LABIREP), Federal University of Ceará, 100, Maurocélio Rocha Ponte Avenue, Sobral 62041-040, CE, Brazil; 4 Microscopy and Microanalysis Laboratory, Department of Cell Biology, Institute of Biological Sciences, University of Brasília, Brasília 70910-900, DF, Brazil; 5 Laboratory of Chemistry of Natural Products, State University of Ceará, 1700, Dr. Silas Munguba Avenue, Fortaleza 60714-903, CE, Brazil; 6 Laboratory of Biochemistry and Gene Expression (LABIEX), Higher Institute of Biomedical Sciences (ISCB), State University of Ceará, 1700, Dr. Silas Munguba Avenue, Fortaleza 60714-903, CE, Brazil

**Keywords:** cancer, antioxidants, pomegranate, cryopreservation, preantral follicles, ovarian tissue

## Abstract

This study aimed to evaluate the effects of an ethanolic extract from *Punica granatum* L. (EE-PG) on bovine ovarian tissue vitrification, focusing on follicular morphology, ultrastructure, stromal cell density, collagen distribution, redox status, and mRNA expression of antioxidant-related genes. Bovine ovarian cortex fragments were divided into a fresh control group for in vivo tissue evaluation or vitrified either with the base vitrification solution (αMEM) alone or supplemented with different concentrations of EE-PG (10, 50, and 100 µg/mL), and subsequently stored in liquid nitrogen for 5 days. After warming, fragments were allocated for morphological and oxidative stress analyses or incubated for 24 h to resumption of cellular metabolism. The concentrations of 10 and 100 µg/mL preserved follicular morphology immediately after warming, and were therefore selected for ultrastructural evaluation. Both concentrations mitigated vitrification-induced damage. Gene expression analysis showed decreased levels of *catalase* (*cat*), *Glutathione Peroxidase 1* (*gpx1*), *and Nuclear Factor Erythroid 2-Related Factor 2* (*nrf2*) compared with the fresh control, whereas Superoxide Dismutase (SOD) enzymatic activity increased after incubation with 10 µg/mL EE-PG compared with all experimental groups. Moreover, Malondialdehyde (MDA) levels in tissues treated with 10 or 100 µg/mL were comparable to fresh controls after incubation. Overall, EE-PG at 10 or 100 µg/mL in the vitrification solution supported the maintenance of tissue morphology, redox balance—despite the downregulation of essential antioxidant genes, which may be associated with a reduced demand for enzymatic antioxidant defense—and cellular metabolism, indicating potential for improving bovine ovarian tissue vitrification outcomes.

## 1. Introduction

Over the last years, the human mortality rate due to pathologies such as cancer has decreased [1], precisely due to the greater effectiveness of new oncological treatments. However, radio- or chemotherapy harms fertility in men and women [2]. Adolescents and young women are particularly impacted by a range of physiological changes, including amenorrhea, ovarian failure, which can lead to early menopause and even the loss of reproductive capacity [3]. In this context, the reproductive biotechnologies, such as ovarian tissue vitrification, emerge as a potential solution to preserve fertility in these women.

Recent advancements in assisted reproduction protocols have contributed to the mitigation of adverse effects associated with caused cancer and its treatment. Ovarian stimulation represents one of the most prevalent procedures in this domain [4] and this procedure allows oocyte and or/embryo cryopreservation. Conversely, for pre-pubertal patients or those requiring immediate treatment, this option may not be feasible due to the time required for stimulation protocols and the immaturity of the reproductive endocrine axis. For these patients, ovarian tissue cryopreservation serves as a viable alternative, given that it ensures the preservation of the ovarian reserve of primordial follicles [5].

Cryopreservation can be performed through slow freezing or vitrification. Vitrification has advantages over slow freezing, mainly because it prevents the formation of intracellular ice crystals [6]. Furthermore, vitrification has become an alternative method since it is a quicker procedure, as well as less expensive [7]. Despite its advantages, the main limitation of vitrification is the fact that it uses high concentrations of cryoprotectants, which possess the potential to induce cellular toxicity [8]. Cytotoxic effects, such as membrane ionic imbalance and organelle degeneration [9], have been demonstrated to render cells more sensitive to reactive oxygen species (ROS) [10]. Given the occurrence of these effects in the ovarian environment, such conditions have the potential to generate DNA damage, thereby compromising follicular and oocyte development [11].

In order to mitigate the aforementioned deleterious effects, several plant-derived substances with antioxidant potential have been investigated. It is well established in the literature that exposure to cryoprotectants increases ROS levels, and, concomitantly, that the addition of antioxidant compounds can help maintain redox balance. Isolated substances, such as anethole, reduce ROS production [12], while essential oils, such as those from *Croton argyrophyllus Kunth,* have been shown to increase thiol levels during vitrification [13], an important marker of oxidative protection. Moreover, such compounds can delay the oxidation of proteins, carbohydrates, lipids, and DNA [14]. Among them, *Punica granatum*, popularly known as pomegranate, has been studied [15]. *Punica granatum* positively influences the expression of *nrf2*. NRF2 is a key factor that regulates oxidative stress [16]. Some studies have shown that *Punica granatum* juice added to vitrification solutions could maintain viability and plasma membrane integrity of bovine semen [17] and buffalo sperm [18]. These beneficial effects are attributed to the main compounds, such punicalagins [19]. Recently, our team has shown that ethanolic extract of *Punica granatum* L preserved that follicular morphology after in vitro culture of bovine ovarian tissue [20]. Based on this, we hypothesized that the *Punica granatum* extract could also exert positive effects during the vitrification of bovine ovarian tissue by preserving its morphological integrity and assisting in the functional recovery of cellular metabolism. The aims of this study were to evaluate the effects of the EE-PG in the vitrification solution of bovine ovarian tissue on (1) follicular morphology and ultrastructure, (2) stromal cell density and extracellular matrix, (3) antioxidant gene expression (nrf2, sod, cat, gpx1) and enzymatic activity, including thiol, MDA, and nitrite levels, and (4) the total antioxidant capacity of the extract.

## 2. Results

### 2.1. Morphological Evaluation, In Vitro Growth, and Stroma Cell Density After Vitrification and 24 h Incubation of Bovine Ovarian Tissue by Histology

**Figure 1:**
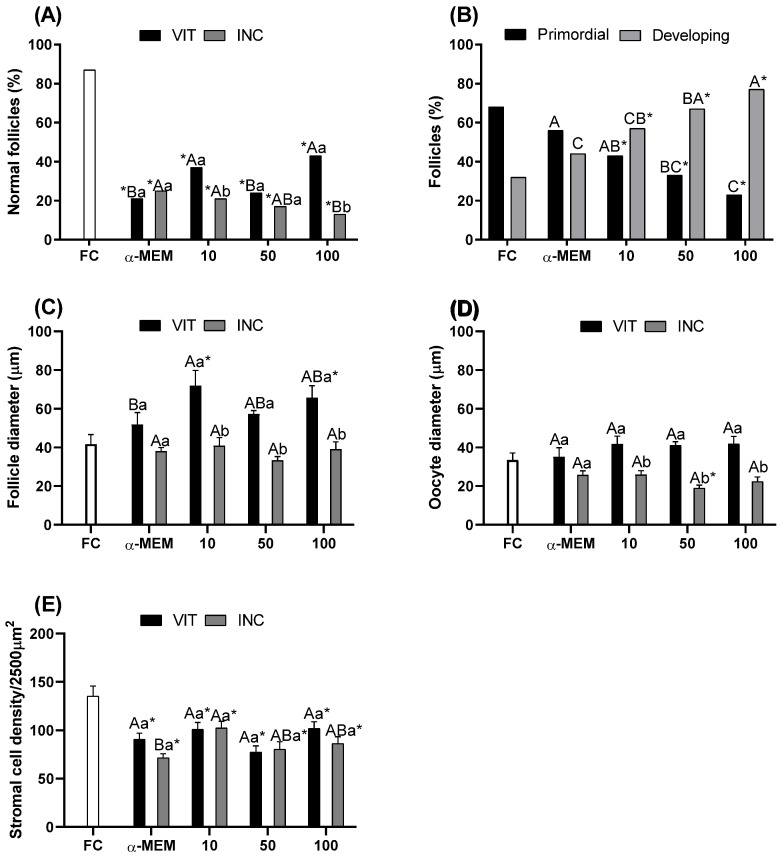
Percentage of morphological normal follicles (**A**), primordial and development follicles (**B**), follicle (**C**) and oocyte (**D**) diameters; and stroma density area (**E**) in fresh bovine ovarian tissue (fresh control, FC) or cryopreserved ovarian tissue in the absence (α-MEM) or presence of EE-PG at 10, 50, or 100 µg/mL immediately after vitrification (VIT) or after vitrification followed by incubation for 24 h (INC). Data are expressed as mean ± SEM from n = 5 animals. All different superscripts differ significantly (*p* < 0.05). * Significantly different from non-vitrified ovarian tissue (fresh control). ^A,B,C^ Different letters denote significant differences among treatments in the same period. ^a,b^ Different letters denote significant differences between time periods (VIT vs. INC) within the same medium.

Figure 1 demonstrates the percentage of morphological normal follicles (Figure 1A), primordial and development follicles (Figure 1B), and stromal cells density (Figure 1C). It can be observed that the percentage of morphological normal follicles (Figure 1A) was significantly decreased (*p* < 0.05) in all treatments after vitrification or after vitrification followed by incubation for 24 h when compared to fresh control. When comparing treatments only after vitrification, tissue exposure to 10 or 100 µg/mL of EE-PG presented a higher percentage of morphologically normal follicles than other treatments; however, after vitrification and 24 h incubation, 100 µg/mL was significantly lower (*p* < 0.05) than 10 µg/mL. Regarding follicle activation after vitrification and 24 h incubation, Figure 1B demonstrates that exposure to both concentrations of EE-PG reduced the percentage of primordial follicles and increased the percentage of developing follicles when compared to fresh control (*p* < 0.05). Moreover, the treatment using 100 µg/mL of EE-PG had a higher (*p* < 0.05) percentage of developing follicles when compared to 10 µg/mL of EE-PG and αMEM.

Both concentrations, 10 and 100 µg/mL, increased (*p* < 0.05) follicular diameter after vitrification compared to fresh control, while only 10 µg/mL was higher than αMEM (Figure 1C). When comparing ovarian tissue that was only vitrified to that which was vitrified followed by incubation for 24 h, it was observed that all EE-PG concentrations tested decreased follicle diameter (*p* < 0.05). Regarding oocyte diameter (Figure 1D), no changes were observed. Stromal cell density was lower with EE-PG treatment than fresh control (Figure 1E). In addition, although there was no difference among treatments after vitrification, after vitrification followed by incubation for 24 h, 10 µg/mL of EE-PG increased (*p* < 0.05) stromal cell density when compared to αMEM, but was not different from 100 µg/mL of EE-PG.

### 2.2. Ultrastructural Morphology After Vitrification and 24 h Incubation of Bovine Ovarian Tissue

**Figure 2:**
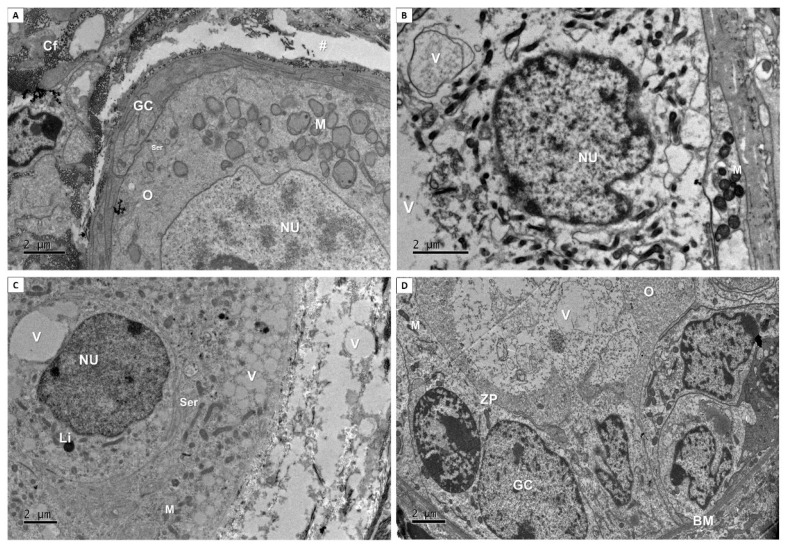
Ultrastructural morphology of preantral follicles from fresh control (**A**), αMEM (**B**), 10 µg/mL (**C**) and 100 µg/mL (**D**) of EE-PG after vitrification followed by incubation for 24 h. Oocyte (O), granulosa cells (GC), nucleus (NU), collagen fibers (Cf), smooth endoplasmic reticulum (Ser), mitochondria (M), vacuole (V), lipid droplet (Li), zone pellucida (ZP), basal membrane (BM), detachment (#) between basal membrane and stroma cells.

For better evaluation of follicular quality, Figure 2 shows ultrastructural analysis that was performed from fresh control (Figure 2A), as well as from αMEM after vitrification (Figure 2B) and from 10 (Figure 2C) and 100 µg/mL (Figure 2D) of EE-PG after vitrification followed by incubation for 24 h. A normal follicle can be seen in fresh control (Figure 2A), in which the integrity of follicular compartments, i.e., granulosa cells and oocyte, was confirmed according to previous histological analysis. In addition, a detachment between stroma cells and follicle basal membrane was observed. Collagen fibers can be seen in this space. Moreover, there were no vacuoles, and organelles were preserved and well distributed. After vitrification, in the αMEM (Figure 2B), a degenerated follicle was observed containing low density of organelles and large vacuoles. After vitrification following incubation for 24 h using 10 (Figure 2C) and 100 µg/mL of EE-PG (Figure 2D), follicle ultrastructural integrity was preserved despite follicles presenting some vacuoles. A follicle with well-defined mitochondria and endoplasmic reticulum was also observed. Interestingly, 100 µg/mL of EE-PG maintained the presence of cuboidal granulosa cells, indicating that this follicle was activated during the 24 h incubation period.

### 2.3. Total Antioxidant Activity of Ethanolic Extract of Punica Granatum (EE-PG)

**Figure 3:**
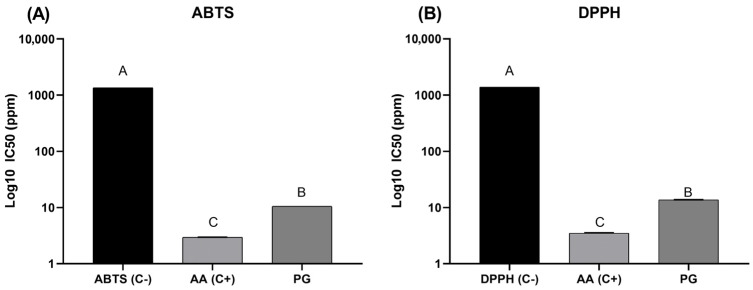
Total antioxidant activity from EE-PG by (**A**) ABTS e (**B**) DPPH. Data are expressed as mean ± SEM from triplicate measurements. ^A,B,C^ Different letters denote significant differences among negative (C-), positive (Ascorbic acid, AA) control, and EE-PG (*p* < 0.05).

The total antioxidant activity of pure EE-PG is depicted in Figure 3. It was observed that EE-PG has a higher (*p* < 0.05) antioxidant capacity than ascorbic acid as a control based on kidnapping of both 2,2-diphenyl-1-picrylhydrazyl (DPPH) (Figure 3A) and 2,2′-azinobis(3-ethylbenzothiazoline-6-sulfonic acid) (ABTS) (Figure 3B).

### 2.4. Extracellular Matrix (ECM) Compounds After Vitrification and 24 h Incubation of Bovine Ovarian Tissue

**Figure 4:**
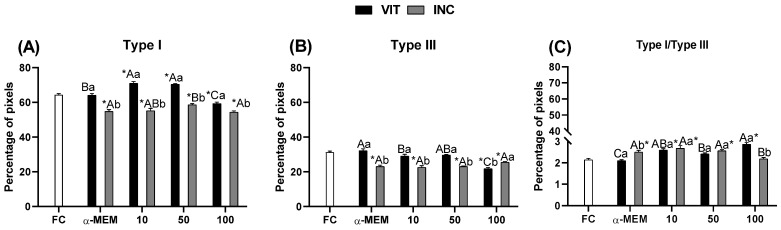
Percentage of pixels for type I (**A**), type III (**B**) and the ratio of type I/type III (**C**) collagen fibers in fresh bovine ovarian tissue (fresh control, FC) or cryopreserved ovarian tissue in the absence (αMEM) or presence of EE-PG at 10, 50, or 100 µg/mL immediately after vitrification (VIT) or after vitrification followed by incubation for 24 h (INC). Data are expressed as mean ± SEM from n = 5 animals. All different superscripts differ significantly (*p* < 0.05). * Significantly different from non-vitrified ovarian tissue (fresh control). ^A,B,C^ Different letters denote significant differences among treatments in the same period. ^a,b^ Different letters denote significant differences between time periods (VIT vs. INC) within the same medium.

Figure 4 shows the collagen type I (Figure 4A) and type III fibers (Figure 4B) and ratio of type I/Type III (Figure 4C) after vitrification and vitrification followed by incubation for 24 h of ovarian tissue using EE-PG. After vitrification only, the percentage of type I collagen fiber (Figure 4A) was increased (*p* < 0.05) in all treatments using EE-PG when compared to fresh control. On the other hand, after vitrification followed by incubation for 24 h, the opposite was observed. All treatments decreased (*p* < 0.05) both type I (Figure 4A) and type III (Figure 4B) collagen fibers when compared to fresh control. Moreover, after vitrification, 10 µg/mL of EE-PG demonstrated the highest (*p* < 0.05) percentage of type I collagen fiber relative to 100 µg/mL and αMEM treatments; meanwhile, for type III collagen fiber, 10 µg/mL of EE-PG was lower (*p* < 0.05) than αMEM treatment. When comparing the same treatment between periods, 10 µg/mL of EE-PG decreases (*p* < 0.05) the percentage of type I and type III collagen fibers after vitrification and 24 h incubation. Evaluation of the collagen fiber ratio (Figure 4C) revealed an increase at concentrations of 10 and 100 µg/mL (*p* < 0.05) after vitrification. After tissue vitrification followed by 24 h of incubation, 100 µg/mL of extract returned the collagen fiber ratio to its initial state, not differing from the fresh control.

### 2.5. Antioxidant Status by Measurement of SOD, CAT and GSH-Px Activities

**Figure 5:**
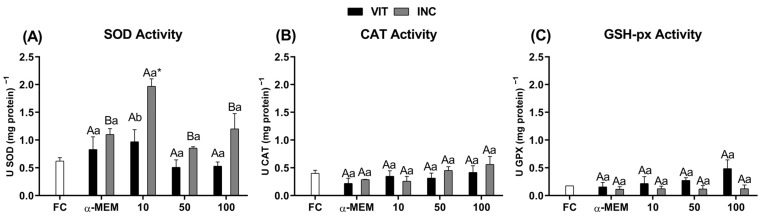
Antioxidant enzyme activity of (**A**) superoxide dismutase (SOD), (**B**) catalase (CAT) and (**C**) glutathione peroxidase (GSH-Px) in fresh bovine ovarian tissue (fresh control, FC) or cryopreserved ovarian tissue in the absence (αMEM) or presence of EE-PG at 10, 50, or 100 µg/mL immediately after vitrification (VIT) or after vitrification followed by incubation for 24 h (INC). Data are expressed as mean ± SEM from n = 3 animals. All different superscripts differ significantly (*p* < 0.05). * Significantly different from non-vitrified ovarian tissue (fresh control). ^A,B^ Different letters denote significant differences among treatments in the same period. ^a,b^ Different letters denote significant differences between time periods (VIT vs. INC) within the same medium.

Antioxidant enzymes activities are demonstrated in Figure 5. It was observed that SOD activity (Figure 5A) was increased (*p* < 0.05) by 10 µg/mL of EE-PG after vitrification followed by incubation for 24 h when compared to fresh control and when compared to this same concentration after only vitrification and warming. On the other hand, ethanolic extract from *Punica granatum* was unable to interfere in CAT (Figure 5B) or GSH-Px (Figure 5C) activities.

### 2.6. Prooxidant Status by Measurement of Nitrite (NO_2_^−^), Malondialdehyde (MDA) and Thiol Levels

**Figure 6:**
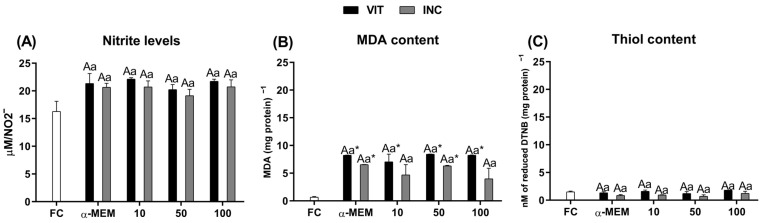
Levels of NO_2_^−^ (**A**), MDA (**B**) and thiol (**C**) in fresh bovine ovarian tissue (fresh control, FC) or cryopreserved ovarian tissue in the absence (αMEM) or presence of EE-PG at 10, 50, or 100 µg/mL immediately after vitrification (VIT) or after vitrification followed by incubation for 24 h (INC). Data are expressed as mean ± SEM from n = 3 animals. All different superscripts differ significantly (*p* < 0.05). * Significantly different from non-vitrified ovarian tissue (fresh control). ^A,B^ Different letters denote significant differences among treatments in the same period. ^a,b^ Different letters denote significant differences between time periods (VIT vs. INC) within the same medium.

Figure 6 demonstrates that nitrite (Figure 6A) and thiol levels (Figure 6C) were similar among treatments, including fresh control, while MDA levels were increased after vitrification in all treatments (*p* < 0.05). Alternatively, MDA levels after vitrification followed by incubation for 24 h were similar to fresh control only in 10 and 100 µg/mL of EE-PG.

### 2.7. mRNA Levels of Cat, Sod, gpx1 and nrf2 After Vitrification Followed by Incubation for 24 h

**Figure 7:**
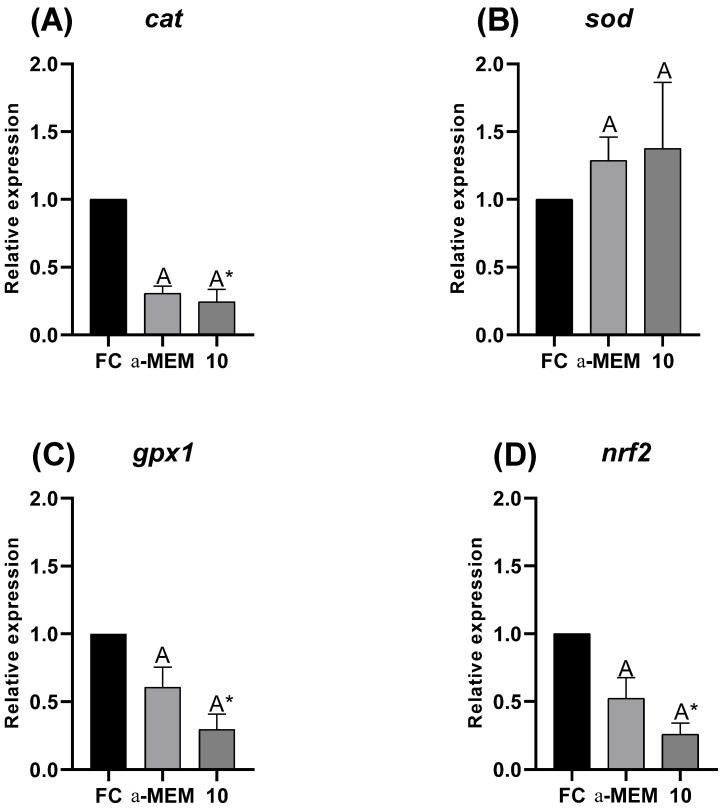
mRNA quantification of cat (**A**), sod (**B**), gpx1 (**C**), and nrf2 (**D**) from fresh control (FC), αMEM, and 10 µg/mL of EE-PG after vitrification followed by incubation for 24 h (INC). Data are expressed as mean ± SEM from n = 4 animals. All different superscripts differ significantly (*p* < 0.05). * Significantly different from non-vitrified ovarian tissue (fresh control). ^A,B^ Different letters denote significant differences between αMEM and 10 µg/mL of EE-PG.

Figure 7 demonstrates that mRNA levels of *cat* (Figure 7A), *gpx1* (Figure 7C) and *nrf2* (Figure 7D) were decreased (*p* < 0.05) in 10 µg/mL of EE-PG when compared to fresh control, except no difference was observed for *sod* (Figure 7B). In addition, when comparing treatments to each other, i.e., 10 µg/mL of EE-PG and αMEM after vitrification and 24 h incubation, there was no difference for all genes evaluated.

## 3. Discussion

The present study described for the first time the effects of EE-PG during bovine ovarian tissue vitrification. It was observed that after vitrification followed by incubation for 24 h, 10 and 100 µg/mL of EE-PG preserved normal follicular morphological ultrastructure showing well-preserved organelles and reduced vacuole formation. The tissue preservation may be due to intracellular repair processes that occur under stress conditions, such as autophagy, which act on the degradation and recycling of damaged organelles [21]. Moreover, the presence of a good density of organelles with normal morphology, such as mitochondria, besides confirming autophagy, highlight the resumption of cellular metabolism. These results in, additionally, the increase in SOD activity, as well as the confirmation of the antioxidant potential of the extract, supporting the concept that EE-PG acted as a powerful antioxidant for follicle preservation.

As mentioned before, the EE-PG has a powerful antioxidant potential confirmed by the ABTS and DPPH radical capture. Corroborating the present study, Jacob et al. [22] verified that different extracts from *Punica granatum* peel have a high antioxidant activity. This antioxidant activity has been attributed to the major compounds presents in the extract, such as punicalagin. In its isolated form, punicalagin presents antiapoptotic and antioxidant effects in cardiomyocytes (H9c2) [23], as well as in fetal neurons (Pc12) [24] cultured in vitro. Moreover, our team recently observed that the addition of EE-PG or punicalagin preserved preantral follicles after in vitro culture [20,25]. Therefore, using *Punica granatum* extracts or its isolated compounds in different steps of the vitrification process may be a promising improvement due to its antioxidant characteristics.

One of the steps of vitrification is the warming process. During this step, reintroduction of the oxygen into the cells occurs, triggering oxidation–reduction reactions, which leads to free radical formation [26] and cell damage. Allied to this, the vitrification process makes follicles less tolerant to their in vitro environment and more predisposed to death [27]. Therefore, an interesting alternative to maintain follicle viability would be the use of the substances capable of mitigating the atresia and ROS production during in vitro incubation period. Carvalho et al. [28] demonstrated that the addition of antioxidant substances to the vitrification and/or warming solution may promote the reduction in ROS. In the present study, we verified that damage caused by vitrification seems to be prevented by EE-PG, since vitrified follicles using 10 and 100 µg/mL of EE-PG exhibited follicle morphology maintenance and increased follicular diameters after warming. Thus, EE-PG can improve different steps of the cryopreservation protocols.

Even after only 24 h of in vitro culture, 100 µg/mL of EE-PG not only preserved follicles but also increased the number of developing follicles. These results are consistent with those that we have previously reported after in vitro culture of bovine preantral follicles using 100 µg/mL of EE-PG (Oliveira et al. [20]). This highlights the activation process signals in which primordial follicles change granulosa cell morphology from flattened to cuboidal cells, and, here, it was confirmed by TEM. The activation process in mammalian is regulated by some signaling pathways, such as fosfatidilinositol-3-quinase/protein kinase B/forkhead box O3a (PI3K-AKT-FOXO3a) and mTORC1 (mechanistic target of rapamycin complex 1) [29]. PI3K-AKT-FOXO3a pathway maintains primordial follicle quiescence, while mTORC1 promotes the activation of these follicles and keeps them viable [28]. In our experiment, the use of the higher concentrations of EE-PG possibly upregulated these pathways in vitrified bovine ovarian follicles. However, studies using a methanolic extract from the flowers of the *Punica granatum* observed the inhibition of the PI3K/AKT phosphorylation in preadipocyte cells (3T3-L1) in a concentration-dependent way [30]. Similarly, Banerjee et al. [31] verified that pomegranate juice also blocked this pathway in a carcinogenesis model. Such variations in the activity of the *Punica granatum* extracts may be due to the different parts of the plant, as well as the way in which the extract is produced. The activation of these pathways is crucial for the successful restoration of gonadal function in oncological patients, as the primary objective of cryopreserved tissue is to resume follicular growth, thereby maintaining hormonal axis function and enabling the possibility of natural conception or conception assisted by reproductive technologies.

To maintain cell–cell interaction and follicle migration within the ovary, it is important to maintain extracellular matrix (ECM) from ovarian stroma cells [32]. Ovarian stromal cells provide support to follicles and together with thecal cells assist the biosynthesis of the estradiol, as well as cell proliferation and apoptosis inhibition [33], and ensure follicle development and oocyte maturation. However, physical processes intrinsic to vitrification may lead to a decrease in the stroma cell density [34]. And, in damage situations with a number or quality reduction in these cells, the ECM has to be constantly and coordinately remodeled. Failures during this remodeling may cause high neovascularization, leading to tissue fibrosis [35]. Fibrosis, in turn, is characterized by the excess of type I collagen and decrease in the type III collagen [36] and may manifest itself as a result of cryopreservation process [37]. In the present study, after 24 h of incubation, 100 µg/mL of EE-PG reduced the type I collagen fibers when compared to 50 µg/mL of EE-PG, maintaining the type III collagen fibers. It is important to highlight that 100 µg/mL of EE-PG also maintained type I/Type III fiber ratio, similarly to the fresh control. Therefore, EE-PG may promote readaptation of the ovarian tissue to the damage process by maintaining the stromal density and avoiding the fibrosis.

Vitrification increases ROS production, leading to oxidative stress [38], which is characterized by the imbalance between pro- and antioxidants agents. This process can alter the activity of the antioxidant defense system, which is composed of SOD, CAT and GSH-Px enzymes. In the present study, after incubation period, the use of 10 µg/mL reduced the expression of mRNA to *nrf2*, as well as to *cat* and *gpx1* enzymes, without altering *sod* mRNA levels. On the other hand, SOD activity was increased, while CAT and GSH-Px activities, as well as MDA levels did not present any changes. Similar effects were observed by Singh et al. [39] and Naghizadeh-Baghi et al. [40]. In the first study, pretreatment using methanolic extract from the *Punica granatum* peel promoted an increase in SOD activity and decrease in MDA levels in the hepatic toxicity model [39]. The same occurred in individuals who practice intense physical exercise after ingesting pomegranate juice [40]. The *nrf2* gene upregulates antioxidant enzymes, such as GSH-Px, CAT and SOD [41]. SOD acts in the conversion of the superoxide anion into a less harmful product, oxygen peroxide, and in the downregulation of lipid peroxidation [42]. Lipid peroxidation [43] is a result of dysfunctional mitochondria, which increase the production of reactive oxygen species (ROS) [44]. In our study, this less oxidative environment may be due to a lower requirement for free radical neutralization which was supported by the maintenance of mitochondrial density and morphology. Together, these results suggest the direct protective effect by *Punica granatum* compounds, which have the capacity to prevent free radical production, reducing the expression of genes involved in the antioxidant response.

In the ovary, nitric oxide (NO) production may occur not only in the follicle cells but in the ovarian vessels also [45]. The presence of this molecule may play a protective effect, reduce granulosa cell apoptosis [46], and control follicle survival [45]. In physiological conditions, NO oxidation can lead to nitrate (NO_3_^−^) and nitrite (NO_2_^−^) formation; thus, their measurements are considered an indirect method to estimate NO concentration. In our study, all treatments were able to maintain nitrite and thiol levels similar to fresh control. The thiol group, -SH, present in the glutathione reduced molecule (GSH), is one of the agents involved in several biological functions, including the reduction in the cytotoxic effects caused by excessive ROS production. The quantity of thiol levels is considered an indirect marker to the antioxidant capacity, since GSH-Px oxidizes GSH into oxidized glutathione (GSSG) and water in the H_2_O_2_ presence. Therefore, the higher thiol content, the lower the H_2_O_2_ content in the sample. In this context, the use of such markers contributes to the elucidation of how antioxidant substances may act on cryopreserved tissue. However, further studies are needed to demonstrate the function of vitrified ovarian tissue in vivo to support follicles that can produce competent oocytes, considering the limitations of the in vitro environment for in situ follicular growth. Additionally, more detailed molecular and proteomic analyses should be considered in future studies, as elucidating the signaling mechanisms through which EE-PG acts on fresh, cultured and/or cryopreserved ovarian follicles will be the topic of future studies and will contribute to improvement of vitrification protocols for ovarian tissue.

## 4. Materials and Methods

### 4.1. Chemicals and Ethical Approval

Unless mentioned otherwise, the chemical reagents were purchased from Sigma Chemical Co. (St Louis, MO, USA). This study was approved by the State University of Ceará (UECE) Ethics Committee for Use of Animals (CEUA/UECE nº 31032.002523/2023-41).

### 4.2. Production of the Ethanolic Extract from Punica Granatum (EE-PG)

The peel of the fruit of *Punica granatum* was removed from fresh and ripe fruits from the local market. The fruit was sanitized, and then, peels were isolated. To carry out the ethanolic extraction, 150 g of the peel was placed in P.A. ethanol for 5 days. After extraction, the solution was taken to a rotary evaporator to remove the solvent [47]. After that, the ethanolic extract from *Punica granatum* (EE-PG) was freeze-dried and stored at room temperature. For experiments, the EE-PG was diluted in ultrapure water at 10, 50 and 100 µg/mL concentrations [48]. The beneficial effects of these concentrations were previously confirmed by our group using in vitro culture of bovine ovarian tissue [20]. The presence of major compounds, such as punicalagin, was identified in this extract, as described by Oliveira et al. [20].

### 4.3. Source of Bovine Ovaries

The ovaries (n = 24) from 12 adult mixed breed cattle were collected at a local slaughterhouse (Sobral, CE, Brazil). The ovaries were washed in 70% alcohol for 10 s, followed by two washes in saline solution containing 0.9% of 100 µg/mL of penicillin and 100 µg/mL of streptomycin. The ovaries were placed into tubes containing 20 mL of saline solution with antibiotics and transported to the laboratory at 4 °C within 1 h [49].

### 4.4. Vitrification and Warming of Bovine Ovarian Tissue

The vitrification and warming were made as described by Lunardi et al. [49] with modifications. Initially, 2 per treatment group fragments were placed in 2 mL of vitrification solution composed by *α*MEM supplemented with 10% fetal bovine serum (FBS), 0.25 M of sucrose, 10% dimethyl sulfoxide (Me_2_SO) and different concentrations of EE-PG (10, 50 and 100 µg/mL). After a 5 min exposure to vitrification solution, fragments underwent solid-surface vitrification on the surface of a metal cube floating in liquid nitrogen (LN2). After this, the vitrified fragments were transferred (with LN2 cooled forceps) into cryovials for storage in LN2 as previously described by Lunardi et al. [49].

For warming, after 5 days of cryostorage, all fragments were removed from LN2, kept at room temperature (RT) (∼25 °C) for 1 min, and then immersed in a water bath at 37 °C (∼1–2 min). The cryoprotectant was removed from ovarian cortex fragments in three step washes containing *α*MEM supplemented with 10% FBS and decreasing concentrations of sucrose (SUC) (0.5 M, 0.25 M, and no SUC, respectively) for 5 min each. The efficiency of the EE-PG for the preservation of preantral follicles was evaluated after warming and after 24 h of in vitro incubation.

### 4.5. In Vitro Incubation of Vitrified Bovine Ovarian Cortex

To evaluate the resumption of metabolism capacity after vitrification and warming, 2 fragments per treatment were incubated in 24-well culture dishes containing 1 mL of culture medium for 24 h. The basic culture medium consisted of αMEM (pH 7.2–7.4) supplemented with bovine serum albumin (BSA; 1.25 mg/mL), glutamine (2 mM), hypoxanthine (2 mM), penicillin/streptomycin (100 µg/mL), and ITS (10 µg/mL of insulin, 5.5 µg/mL of transferrin, and 10 µg/mL of selenium). The culture media was previously incubated for 2 h for stabilization of pH and temperature. Culture was performed at 38.5 °C in 5% CO_2_ in a humidified incubator. The incubation period and medium composition were based on a previous study [50].

### 4.6. Morphological and Follicular Development Analysis by Histology

Morphological evaluations were conducted using ovaries collected from five animals. Ovarian samples were analyzed both before vitrification (fresh control) and after vitrification or incubation for 24 h. Tissues were fixed in 4% paraformaldehyde prepared in PBS for 12 h, followed by dehydration, according to standard histological processing procedures. Subsequently, samples were embedded, sectioned at 7 µm thickness, and stained with hematoxylin and eosin. Follicles were classified according to their developmental stage as primordial (oocyte surrounded by a single layer of flattened granulosa cells) or growing follicles, including primary follicles (oocyte surrounded by one layer of cuboidal granulosa cells) and secondary follicles (oocyte surrounded by two or more layers of cuboidal granulosa cells), following established criteria [49]. Each follicle was individually evaluated and considered histologically normal when presenting an intact oocyte without cytoplasmic retraction or nuclear pyknosis, surrounded by well-organized granulosa cells arranged in one or more layers and lacking pyknotic nuclei. Follicles showing oocyte retraction, nuclear pyknosis, and/or disorganization or detachment of granulosa cells from the basement membrane were classified as atretic. A total of 150 follicles were assessed per treatment, corresponding to 30 follicles per animal. The proportions of morphologically normal primordial and growing follicles were calculated for both fresh control samples and post-culture conditions. In addition, follicular diameters were measured after warming followed by 24 h of incubation.

### 4.7. Bovine Ovarian Stromal Cell Density Evaluation

The density of ovarian stromal cells was determined by manually counting cell nuclei in histological sections, as previously described [51]. For each experimental group (fresh control, αMEM, and 10, 50, and 100 µg/mL of EE-PG), 30 sections were analyzed. In each selected section, four random microscopic fields (50 × 50 µm; total area of 2500 µm^2^ per field) were captured to calculate the mean stromal cell density. Images were obtained using a DS cooled camera head (DS-Ri1) coupled to a light microscope (Nikon Eclipse 80i, Nikon, Tokyo, Japan) at 400× magnification. Quantitative analyses were performed using ImageJ software (version 1.54k). To ensure consistency and minimize variability, all counts and measurements were carried out by the same trained operator.

### 4.8. Ultrastructural Morphology of Preantral Follicles Present in Vitrified Bovine Ovarian Tissue by Transmission Electronic Microscopy (TEM)

To assess follicular ultrastructural morphology, ovarian tissue fragments were selected from the fresh control group, αMEM alone (used as an in vitro environment control), and from tissues treated with 10 or 100 µg/mL of EE-PG after vitrification followed by 24 h of incubation. These groups were chosen based on the histological outcomes. Ovarian samples were cut into small fragments (~1 mm^3^) and initially fixed for 24 h at room temperature in a solution containing 2% paraformaldehyde and 2.0% glutaraldehyde prepared in 0.1 M sodium cacodylate buffer (pH 7.2). Following primary fixation, the samples were rinsed in the same buffer and subsequently post-fixed for 1 h in a mixture of 2% osmium tetroxide and 1.6% potassium ferricyanide diluted in 0.2 M sodium cacodylate buffer. En bloc contrast was then performed using an aqueous solution of 0.5% uranyl acetate for 2 h. The tissues were dehydrated through an ascending acetone series (30–100%) and embedded in Spurr resin. Semi-thin sections (3 µm thick) were obtained using an ultramicrotome Leica EM UC7 (Leica Microsystems, Vienna, Austria), stained with toluidine blue, and examined by light microscopy. Ultrathin sections (60–70 nm) were contrasted with uranyl acetate and lead citrate and analyzed using a transmission electron microscope JEOL 1011 (JEOL Ltd., 1011, Tokyo, Japan). Ultrastructural evaluation included analysis of organelle density and preservation, cytoplasmic vacuolization, basement membrane integrity, and granulosa cell organization, as previously described [49].

### 4.9. Evaluation of the Total Antioxidant Capacity of Ethanolic Extract of Punica Granatum

The antioxidant capacity of the ethanolic extract of *Punica granatum* was evaluated using assays based on the free radical capture of 2,2-azinobis(3-ethylbenzothiazoline-6-sulfonic acid) (ABTS) and 2,2-diphenyl-1-picrylhydrazyl (DPPH). These assays were conducted following the protocol described by Do Nascimento et al. [47], with minor adaptations.

#### 4.9.1. ABTS Radical Scavenging

For the ABTS assay, aliquots of 15, 30, and 60 µL of the extract stock solution (200 µg/mL) were added to test tubes containing 3 mL of a previously prepared ABTS radical solution, yielding final extract concentrations of 1, 2, and 4 µg/mL. The reaction mixtures were gently agitated and incubated for 6 min. Absorbance was then measured using a UV–visible spectrophotometer at 734 nm. The antioxidant activity was calculated as the percentage of radical capture and expressed as the concentration required to inhibit 50% of the radical activity (CE_50_, µg/mL). All measurements were performed in triplicate and independently repeated three times. For each assay, a calibration curve was generated, presenting a correlation coefficient R ± 0.995.

#### 4.9.2. DPPH Radical Scavenging

The DPPH assay was carried out under reduced light conditions to prevent photo-degradation of the reagent. Aliquots of 10, 20, 40, and 80 µL of the extract stock solution (200 µg/mL) were mixed with 1990, 1980, 1960, and 1920 µL of a 0.06 mM methanolic DPPH solution, respectively, resulting in final extract concentrations of 1, 2, 4, and 8 µg/mL. Each condition was tested in triplicate and repeated under identical experimental conditions. Absorbance readings were obtained at 517 nm using a UV spectrophotometer, recorded at one-minute intervals until stabilization of the absorbance values. Methanol was used as the blank, and ascorbic acid served as the positive control. The antioxidant capacity was expressed as the percentage of radical capture and calculated as CE_50_ (µg/mL of DPPH). Regression analysis was performed individually for each sample, including the dichloromethane fraction, yielding a correlation coefficient of R = 0.999 and the equation Y = 0.7263x − 0.87.

### 4.10. Extracellular Matrix Analysis in Vitrified Bovine Ovarian Tissue

The distribution of collagen fibers within the extracellular matrix was assessed using Picrosirius Red staining (Abcam Kit; Nova Analítica, São Paulo, Brasil). Ovarian tissue sections (7 μm thick) were initially deparaffinized in xylene and subsequently stained with a 0.1% Sirius Red solution for 1 h at room temperature. After staining, excess dye was removed by washing the sections with a 0.5% acetic acid solution. The samples were then dehydrated and permanently mounted on glass slides. Collagen fibers were visualized under polarized light microscopy, allowing differentiation based on birefringence patterns: type I collagen fibers appeared yellow to orange, whereas type III collagen fibers were identified by green birefringence. Images were captured using a polarized light microscope (Nikon Eclipse 80i, Nikon, Tokyo, Japan; 400× magnification) equipped with a digital camera (Nikon DS-Ri1, Nikon, Tokyo, Japan). A total of fifty sections were analyzed for each treatment group. Quantitative analysis of collagen distribution was performed using ImageJ software by applying RGB thresholding to determine the proportion of each birefringent color, expressed as pixel percentages. The ratio between type I and type III collagen fibers was subsequently calculated (type I/type III) to evaluate changes in collagen organization.

### 4.11. Biochemical Analyses of Vitrified Bovine Ovarian Tissue

For biochemical evaluations, the experiments were independently repeated three times using six ovaries. Following vitrification and a 24 h incubation period, ovarian fragments (100 mg/mL) were homogenized in potassium phosphate buffer (KH_2_PO_4_ and K_2_HPO_4_; P9791 and P3786; Sigma-Aldrich, St. Louis, MO, USA) at a 1:9 ratio, supplemented with protease inhibitors (aprotinin, 5 mg/mL, and phenylmethylsulfonyl fluoride—PMSF, 34.8 mg/mL), with the pH adjusted to 7.5. The resulting homogenates were centrifuged at 1500× *g* for 10 min at 4 °C, and the supernatants were collected for subsequent spectrophotometric analyses. All assays were performed using quartz cuvettes (Genesis 10s UV–Vis; Thermo Scientific, Waltham, MA, USA). Results are presented as mean ± standard error of the mean (SEM) and expressed as enzyme activity units per milligram of protein (U/mg protein). Biochemical analyses followed the protocols previously described by De Aguiar et al. [52].

#### 4.11.1. Total Proteins (Bradford Method)

Protein content was quantified by the Bradford assay using Coomassie Brilliant Blue dye (Quick Start™ Bradford Protein Assay; Cat. No. 500–0205; Bio-Rad, Hercules, CA, USA) to determine the total protein concentration in each extract. The Coomassie dye binds to proteins, forming a blue-colored complex whose absorbance is proportional to protein concentration. Absorbance was measured spectrophotometrically at 595 nm. Protein concentrations were calculated based on a standard curve generated with bovine serum albumin (BSA) at concentrations of 0, 2.5, 5, 10, 15, 25, 35, and 50 mg/mL. The resulting protein values were used to normalize pro-oxidant and antioxidant parameters.

#### 4.11.2. Superoxide Dismutase Activity (SOD)

Superoxide dismutase activity was determined based on the inhibition of adrenaline autoxidation in an alkaline medium. For the assay, a catalase solution (0.048 mg/mL; C9322; Sigma-Aldrich, St. Louis, MO, USA) was prepared and added to glycine buffer (7:3, pH 10.2; Dinâmica, São Paulo, Brazil). Subsequently, the ovarian homogenate was added, followed by adrenaline solution (0.218 mg/mL; E4260; Sigma-Aldrich, St. Louis, MO, USA) to initiate the oxidation reaction. Changes in absorbance were monitored at 480 nm at 10 s intervals for a total duration of 180 s.

#### 4.11.3. Catalase Activity (CAT)

Catalase activity was assessed by monitoring the rate of hydrogen peroxide (H_2_O_2_) decomposition. The reaction mixture consisted of H_2_O_2_ solution (950 μg/mL; PH09717RA; Êxodo Científica, Sumaré, SP, Brazil) prepared in phosphate-buffered saline (PBS; pH 7.4) at room temperature and placed in a quartz cuvette. The reaction was initiated by the addition of ovarian homogenate. The decrease in H_2_O_2_ concentration was recorded spectrophotometrically at 240 nm, with measurements taken every 30 s, in duplicate.

#### 4.11.4. Glutathione Peroxidase Activity (GSH-Px)

Glutathione peroxidase activity was determined by following the oxidation of NADPH. In this coupled assay, NADPH is consumed by glutathione reductase (GR; G3664; Sigma-Aldrich, St. Louis, MO, USA) to regenerate reduced glutathione (GSH) from oxidized glutathione (GSSG). In the presence of GSH-Px and hydrogen peroxide, GSH is oxidized to GSSG while peroxides are reduced to water. Thus, the rate of NADPH consumption is directly proportional to GSH-Px activity. The reaction mixture consisted of 500 μL of PBS buffer supplemented with EDTA, GR (38 μg/mL), and GSH (3 μg/mL), to which 100 μL of ovarian homogenate was added and incubated for 10 min to allow interaction among GSH-Px, GR, and GSH. Subsequently, 100 μL of NADPH and 100 μL of H_2_O_2_ were added to initiate the reaction. The decrease in absorbance was monitored at 340 nm every 10 s for 300 a.

#### 4.11.5. Determination of Nitrite Levels (NO_2_^−^)

Nitrite concentration was assessed in duplicate by means of the Griess reaction. Briefly, aliquots of ovarian homogenate (100 µL) were mixed with an equal volume of Griess reagent, composed of 1% sulfanilamide prepared in 5% phosphoric acid and 0.1% N-(1-naphthyl) ethylenediamine dihydrochloride (NEED) in distilled water, combined in a 1:1:1:1 ratio. Quantification was performed using a calibration curve generated from serial dilutions of sodium nitrite (NaNO_2_). Absorbance readings were recorded at 540 nm, and nitrite levels were expressed as micromolar (µM) concentrations.

#### 4.11.6. Determination of the MDA Levels by Production of Thiobarbituric Acid Reactive Substances (TBARS)

Lipid peroxidation was evaluated by quantifying thiobarbituric acid reactive substances (TBARS), which reflect malondialdehyde (MDA) formation as a terminal product of membrane lipid oxidation. Ovarian tissue homogenate (63 µL) was combined with 100 µL of 35% perchloric acid and centrifuged at 5000 rpm for 10 min at 4 °C. Subsequently, 150 µL of the resulting supernatant was transferred to a new tube and mixed with 50 µL of 1.2% thiobarbituric acid solution. The reaction mixture was incubated at 95 °C for 30 min. Absorbance was measured at 535 nm to determine MDA levels.

#### 4.11.7. Determination of Pro-Oxidative Status Through the Content of Thiol

The total thiol content was used as an indicator of pro-oxidative status and determined using 5,5′-dithiobis-(2-nitrobenzoic acid) (DTNB; Dinâmica D8130, São Paulo, Brazil). Reduced thiol groups react with DTNB (10 µM), resulting in cleavage of the disulfide bond and formation of 2-nitro-5-thiobenzoate (NTB^2−^), which is stable and detectable at neutral pH. The concentration of NTB^2−^ was quantified spectrophotometrically at 412 nm, and results were expressed as nanomoles of reduced DTNB per milligram of protein.

### 4.12. Quantification of mRNA for Cat, Sod, gpx1, and nfr2 in Vitrified Bovine Ovarian Tissue

Gene expression analysis was performed using samples from the experimental condition that showed the most favorable enzymatic activity results. Accordingly, the following groups were evaluated: fresh control, αMEM alone, and αMEM supplemented with 10 µg/mL EE-PG after vitrification followed by 24 h of incubation. This experiment was independently replicated four additional times using ovarian tissue obtained from four different animals. Total RNA extraction was carried out using Trizol^®^ reagent (Invitrogen, São Paulo, Brazil) in accordance with the manufacturer’s protocol. Frozen ovarian samples received 800 µL of Trizol^®^, and tissue disruption was facilitated by repeated aspiration through a 20-gauge needle prior to centrifugation at 10,000× *g* for 3 min at room temperature. The lysates were subsequently diluted 1:1 with 70% ethanol and transferred to the spin columns provided in the purification kit. After RNA binding, genomic DNA contamination was eliminated by treatment with RNase-free DNase (340 K units/mL) for 15 min at room temperature. The columns were then washed three times, and total RNA was eluted using 30 µL of RNase-free water. RNA concentration and purity were determined spectrophotometrically (BioDrop, Cambridge, UK) by measuring absorbance at 260 nm and evaluating the 260/280 nm ratio. For cDNA synthesis, 2.44 µg of total RNA from each sample was used. RNA samples were initially denatured at 70 °C for 5 min and immediately cooled on ice. Reverse transcription was conducted in a final volume of 20 µL containing reverse transcriptase buffer (4 µL), RNA template (10 µL), RNase inhibitor (8 units), Superscript III reverse transcriptase (150 units), 10 mM dithiothreitol, random primers (0.036 U), and 0.5 mM of each dNTP (Invitrogen). The reaction was incubated at 42.1 °C for 1 h, followed by enzyme inactivation at 80 °C for 5 min. Synthesized cDNA was stored at −20 °C until analysis. Negative controls were prepared without the addition of reverse transcriptase. Quantitative real-time PCR was performed using a StepOne Plus™ system (Applied Biosystems, Foster City, CA, USA). Each reaction contained 10 µL of SYBR Green Master Mix, 7.3 µL of ultrapure water, 1 µL of cDNA, and 5 mM of each specific primer. Primer sequences were designed to amplify cat, sod, gpx1, nrf2, and gapdh (reference gene), as listed in Table 1. Specificity of amplification was confirmed by melting curve analysis. Thermal cycling conditions consisted of an initial denaturation and polymerase activation step at 95 °C for 10 min, followed by 40 cycles of denaturation at 95 °C for 15 s, annealing at 58 °C for 30 s, and extension at 72 °C for 30 s. A final extension step was carried out at 72 °C for 10 min. Negative controls lacking cDNA were included in all runs. Relative gene expression levels were calculated using the 2^−ΔΔCt^ method [53].

### 4.13. Statistical Analysis

The results were expressed as the mean of the replicates and their corresponding ± SEM. The Shapiro–Wilk normality test was performed. Qualitative classification data were expressed according to their proportion and evaluated by the chi-square method. Quantitative data were analyzed by ANOVA, followed by Tukey’s test or the Kruskal–Wallis test, depending on data distribution. Statistical analyses were conducted using GraphPad Prism 9.0 (GraphPad Software, Inc., San Diego, CA, USA). For all statistical analyses, *p* < 0.05 was considered significant.

## 5. Conclusions

In summary, the addition of 10 or 100 µg/mL of the ethanolic extract of EE-PG maintained the follicle morphological quality, as well as ovarian stroma density, by reducing ultrastructural changes and maintaining a less oxidizing environment after vitrification process. This condition was associated with a reduced expression of key genes involved in antioxidant pathways, indicating that the activation of these pathways was not required due to the effective scavenging of free radicals by compounds derived from EE-PG. Therefore, EE-PG has potential for improving the vitrification outcomes of bovine ovarian tissue.

## Figures and Tables

**Table 1:** Primer pairs used for real-time PCR.

Sense (S)	Primer Sequence (5′ → 3′)	Sense (S) Anti-Sense (As)	GenBank Accession No.
*gapdh*	TGTTTGTGATGGGCGTGAACCAATG GCGCGTGGACAGTGGTCATAA	S AS	GI: 402744670
*gpx1*	AACGTAGCATCGCTCTGAGG GATGCCCAAACTGGTTGCAG	S AS	GI: 156602645
*sod*	GTGAACAACCTCAACGTCGC GGGTTCTCCACCACCGTTAG	S AS	GI: 31341527
*cat*	AAGTTCTGCATCGCCACTCA GGGGCCCTACTGTCAGACTA	S AS	GI: 402693375
*nrf2*	GACCCAGTCCAACCTTTGTC GACCCGGACTTACAGGTACT	S AS	GI: 0304941

## Data Availability

The original contributions presented in this study are included in the article. Further inquiries can be directed to the corresponding author.

## References

[1] Siegel R.L., Miller K.D., Wagle N.S., Jemal A. (2023). Cancer statistics, 2023. CA Cancer J. Clin..

[2] Del-Pozo-Lérida S., Salvador C., Martínez-Soler F., Tortosa A., Perucho M., Giménez-Bonafé P. (2019). Preservation of fertility in patients with cancer (Review). Oncol. Rep..

[3] Tiong V., Rozita A.M., Taib N.A., Yip C.H., Ng C.H. (2014). Incidence of chemotherapy-induced ovarian failure in premenopausal women undergoing chemotherapy for breast cancer. World J. Surg..

[4] Akel R.A., Guo X.M., Moravek M.B., Confino R., Smith K.N., Lawson A.K., Klock S.C., Iii E.J.T., Pavone M.E. (2020). Ovarian Stimulation is Safe and Effective for Patients with Gynecologic Cancer. J. Adolesc. Young Adult Oncol..

[5] Gamzatova Z., Komlichenko E., Kostareva A., Galagudza M., Ulrikh E., Zubareva T., Sheveleva T., Nezhentseva E., Kalinina E. (2014). Autotransplantation of cryopreserved ovarian tissue--effective method of fertility preservation in cancer patients. Gynecol. Endocrinol..

[6] Lee S., Ryu K.J., Kim B., Kang D., Kim Y.Y., Kim T. (2019). Comparison between Slow Freezing and Vitrification for Human Ovarian Tissue Cryopreservation and Xenotransplantation. Int. J. Mol. Sci..

[7] Isachenko V., Isachenko E., Weiss J.M., Todorov P., Kreienberg R. (2009). Cryobanking of human ovarian tissue for anti-cancer treatment: Comparison of vitrification and conventional freezing. Cryo Lett..

[8] Zhang J.M., Li L.X., Liu X.L., Yang Y.X., Wan X.P. (2009). Sucrose affecting successful transplantation of vitrified-thawed mouse ovarian tissues. J. Assist. Reprod. Genet..

[9] Borges E.N., Silva R.C., Futino D.O., Rocha-Junior C., Amorim C., Báo S., Lucci C. (2009). Cryopreservation of swine ovarian tissue: Effect of different cryoprotectants on the structural preservation of preantral follicle oocytes. Cryobiology.

[10] Guérin P., El Mouatassim S., Ménézo Y. (2001). Oxidative stress and protection against reactive oxygen species in the pre-implantation embryo and its surroundings. Hum. Reprod. Update.

[11] Silva L.M., Mbemya G.T., Guerreiro D.D., Brito D.C.C., Donfack N.J., Morais M.L.G., Rodrigues G.Q., Bruno J.B., Rocha R.M.P., Alves B.G. (2018). Effect of Catalase or Alpha Lipoic Acid Supplementation in the Vitrification Solution of Ovine Ovarian Tissue. Biopreserv Biobank.

[12] Dos Santos Morais M.L.G., de Brito D.C.C., Pinto Y., Silva L.M., Vizcarra D.M., Silva R.F., Cibin F.W.S., Campello C.C., Alves B.G., Araújo V.R. (2019). Natural antioxidants in the vitrification solution improve the ovine ovarian tissue preservation. Reprod. Biol..

[13] De Souza Santana P., Costa F.D.C., Caetano Filho F.F., Bezerra V.S., Silva B.R., Silva A.d.A., Marcelino É.C., Chaves S.C., Aguiar C.E.R., Martins S.D. (2025). Morphological changes in ovarian follicles, stromal cells, and extracellular matrix in cryopreserved cattle ovarian tissue and the beneficial effects of *Croton argyrophyllus Kunth* essential oil. Cryobiology.

[14] Sindhi V., Gupta V., Yadavilli K., Bhatnagar S., Kumari R., Dhaka N. (2013). Potential applications of antioxidants—A review. J. Pharm. Res..

[15] Ammar A., Turki M., Hammouda O., Chtourou H., Trabelsi K., Bouaziz M., Abdelkarim O., Hoekelmann A., Ayadi F., Souissi N. (2017). Effects of Pomegranate Juice Supplementation on Oxidative Stress Biomarkers Following Weightlifting Exercise. Nutrients.

[16] Huang W.C., Liou C.J., Shen S.C., Hu S., Chao J.C.-J., Huang C., Wu S.-J. (2024). Punicalagin from pomegranate ameliorates TNF-α/IFN-γ-induced inflammatory responses in HaCaT cells via regulation of SIRT1/STAT3 axis and Nrf2/HO-1 signaling pathway. Int. Immunopharmacol..

[17] El-Sheshtawy R., Sisy G., El-Nattat W. (2016). Effects of pomegranate juice in Tris-based extender on cattle semen quality after chilling and cryopreservation. Asian Pac. J. Reprod..

[18] Javed M., Tunio M.T., Abdul Rauf H., Bhutta M.F., Naz S., Iqbal S. (2019). Addition of pomegranate juice (*Punica granatum*) in tris-based extender improves post-thaw quality, motion dynamics and in vivo fertility of Nili Ravi buffalo (*Bubalus bubalis*) bull spermatozoa. Andrologia.

[19] Laurindo L.F., Rodrigues V.D., Minniti G., de Carvalho A.C.A., Zutin T.L.M., DeLiberto L.K., Bishayee A., Barbalho S.M. (2024). Pomegranate (*Punica granatum* L.) phytochemicals target the components of metabolic syndrome. J. Nutr. Biochem..

[20] Oliveira M.A.F., Martins S.D., de Assis E.I.T., Martins J.E.R., Alves F.L., Bittencourt S.R.A., da Silva I.G.M., Báo S.N., Fidelis Q.C., de Morais S.M. (2025). Ethanolic Extract of Pomegranate (*Punica granatum* L.) Prevents Oxidative Stress and Preserves the Morphology of Preantral Follicles Included in Bovine Ovarian Tissue Cultured In Vitro. Animals.

[21] Bang S., Shin H., Song H., Suh C.S., Lim H.J. (2014). Autophagic activation in vitrified-warmed mouse oocytes. Reproduction.

[22] Jacob J., Lakshmanapermalsamy P., Illuri R., Bhosle D., Sangli G.K., Mundkinajeddu D. (2018). In vitro Evaluation of Antioxidant Potential of Isolated Compounds and Various Extracts of Peel of *Punica granatum* L.. Pharmacogn. Res..

[23] Ye M., Zhang L., Yan Y., Lin H. (2019). Punicalagin protects H9c2 cardiomyocytes from doxorubicin-induced toxicity through activation of Nrf2/HO-1 signaling. Biosci. Rep..

[24] Clementi M.E., Pani G., Sampaolese B., Tringali G. (2018). Punicalagin reduces H_2_O_2_-induced cytotoxicity and apoptosis in PC12 cells by modulating the levels of reactive oxygen species. Nutr. Neurosci..

[25] Bezerra V.S., Costa F.C., Caetano Filho F.F., Costa J.J.N., Neto M.F.d.L., Furtado C.L.M., Ceccatto V.M., Araújo V.R., Silva J.R.V. (2024). Punicalagin increases follicular activation, development and activity of superoxide dismutase 1, catalase, and glutathione peroxidase 1 in cultured bovine ovarian tissues. Reprod. Fertil. Dev..

[26] Rocha C.D., Soares M.M., de Cássia Antonino D., Júnior J.M., Mohallem R.F.F., Rodrigues A.P.R., Figueiredo J.R., Beletti M.E., Jacomini J.O., Alves B.G. (2018). Positive effect of resveratrol against preantral follicles degeneration after ovarian tissue vitrification. Theriogenology.

[27] Donfack N.J., Alves K.A., Alves B.G., Rocha R.M.P., Bruno J.B., Lima L.F., Lobo C.H., Santos R.R., Domingues S.F.S., Bertolini M. (2018). In vivo and in vitro strategies to support caprine preantral follicle development after ovarian tissue vitrification. Reprod. Fertil. Dev..

[28] Carvalho A.A., Faustino L.R., Silva C.M., Castro S., Lobo C., Santos F., Santos R., Campello C., Bordignon V., Figueiredo J. (2014). Catalase addition to vitrification solutions maintains goat ovarian preantral follicles stability. Res. Vet. Sci..

[29] Reddy P., Zheng W., Liu K. (2010). Mechanisms maintaining the dormancy and survival of mammalian primordial follicles. Trends Endocrinol. Metab..

[30] Li T., Zhang L., Jin C., Xiong Y., Cheng Y.Y., Chen K. (2020). Pomegranate flower extract bidirectionally regulates the proliferation, differentiation and apoptosis of 3T3-L1 cells through regulation of PPARγ expression mediated by PI3K-AKT signaling pathway. Biomed. Pharmacother..

[31] Banerjee N., Kim H., Talcott S., Mertens-Talcott S. (2013). Pomegranate polyphenolics suppressed azoxymethane-induced colorectal aberrant crypt foci and inflammation: Possible role of miR-126/VCAM-1 and miR-126/PI3K/AKT/mTOR. Carcinogenesis.

[32] Woodruff T.K., Shea L.D. (2007). The role of the extracellular matrix in ovarian follicle development. Reprod. Sci..

[33] Qiu M., Liu J., Han C., Wu B., Yang Z., Su F., Quan F., Zhang Y. (2014). The influence of ovarian stromal/theca cells during in vitro culture on steroidogenesis, proliferation and apoptosis of granulosa cells derived from the goat ovary. Reprod. Domest. Anim..

[34] Gastal G.D.A., Aguiar F.L.N., Alves B.G., Alves K., de Tarso S., Ishak G., Cavinder C., Feugang J., Gastal E. (2017). Equine ovarian tissue viability after cryopreservation and in vitro culture. Theriogenology.

[35] Rüger B.M., Buchacher T., Dauber E.M., Pasztorek M., Uhrin P., Fischer M.B., Breuss J.M., Leitner G.C. (2020). De novo Vessel Formation Through Cross-Talk of Blood-Derived Cells and Mesenchymal Stromal Cells in the Absence of Pre-existing Vascular Structures. Front. Bioeng. Biotechnol..

[36] Karsdal M. (2019). Biochemistry of Collagens, Laminins and Elastin: Structure, Function and Biomarkers.

[37] Kitajima M., Murakami N., Kitajima Y., Kajimura I., Matsumura A., Matsumoto K., Harada A., Miura K. (2022). Accumulation of fibrosis and altered perifollicular stromal differentiation in vitrified-thawed human ovarian tissue xenografted to nude mice. Reprod. Med. Biol..

[38] Kaushik K., Gupta P., Johnson P., Krishna K., Nandi S., Mondal S., Tej J.N.K., Bence S., Cseh S. (2022). Effect of retinol in the vitrification medium on viability of vitrified ovine preantral follicles and expression of key developmental and apoptosis related genes. Cryo Lett..

[39] Singh R.P., Chidambara Murthy K.N., Jayaprakasha G.K. (2002). Studies on the antioxidant activity of pomegranate (*Punica granatum*) peel and seed extracts using in vitro models. J. Agric. Food Chem..

[40] Naghizadeh-Baghi A., Mazani M., Shadman-Fard A., Nemati A. (2015). *Punica granatum* juice effects on oxidative stress in severe physical activity. Mater. Sociomed..

[41] Liu X., Ma Y., Luo L., Zong D., Li H., Zeng Z., Cui Y., Meng W., Chen Y. (2022). Dihydroquercetin suppresses cigarette smoke induced ferroptosis in the pathogenesis of chronic obstructive pulmonary disease by activating Nrf2-mediated pathway. Phytomedicine.

[42] Rao Y.L., Ganaraja B., Marathe A., Manjrekar P.A., Joy T., Ullal S., Pai M.M., Murlimanju B.V. (2021). Comparison of malondialdehyde levels and superoxide dismutase activity in resveratrol and resveratrol/donepezil combination treatment groups in Alzheimer’s disease induced rat model. 3 Biotech.

[43] Almeida A.M., Bertoncini C.R., Borecký J., Souza-Pinto N.C., Vercesi A.E. (2006). Mitochondrial DNA damage associated with lipid peroxidation of the mitochondrial membrane induced by Fe^2+^-citrate. An. Acad. Bras. Cienc..

[44] Angelova P.R., Esteras N., Abramov A.Y. (2021). Mitochondria and lipid peroxidation in the mechanism of neurodegeneration: Finding ways for prevention. Med. Res. Rev..

[45] Basini G., Grasselli F. (2015). Nitric oxide in follicle development and oocyte competence. Reproduction.

[46] Chun S.Y., Eisenhauer K.M., Minami S., Billig H., Perlas E., Hsueh A.J. (1996). Hormonal regulation of apoptosis in early antral follicles: Follicle-stimulating hormone as a major survival factor. Endocrinology.

[47] Do Nascimento J.E.T., Rodrigues A.L.M., de Lisboa D.S., Liberato H.R., Falcão M.J.C., Da Silva C.R., Júnior H.V.N., Filho R.B., Junior V.F.D.P., Alves D.R. (2018). Chemical Composition and Antifungal In Vitro and In Silico, Antioxidant, and Anticholinesterase Activities of Extracts and Constituents of Ouratea fieldingiana (DC.) Baill. Evid. Based Complement. Altern. Med..

[48] Seeram N.P., Adams L.S., Henning S.M., Niu Y., Zhang Y., Nair M., Heber D. (2005). In vitro antiproliferative, apoptotic and antioxidant activities of punicalagin, ellagic acid and a total pomegranate tannin extract are enhanced in combination with other polyphenols as found in pomegranate juice. J. Nutr. Biochem..

[49] Lunardi F.O., Araújo V.R., Faustino L.R., Carvalho A.d.A., Gonçalves R.F.B., Bass C.S., Báo S.N., Name K.P.O., Campello C.C., de Figueiredo J.R. (2012). Morphologic, viability and ultrastructural analysis of vitrified sheep preantral follicles enclosed in ovarian tissue. Small Rumin. Res..

[50] Carvalho A.A., Faustino L.R., Silva C.M., Castro S., Luz H., Rossetto R., Lopes C., Campello C., Figueiredo J., Rodrigues A. (2011). Influence of vitrification techniques and solutions on the morphology and survival of preantral follicles after in vitro culture of caprine ovarian tissue. Theriogenology.

[51] Alves K.A., Alves B.G., Gastal G.D., de Tarso S.G.S., Gastal M.O., Figueiredo J.R., Gambarini M.L., Gastal E.L. (2016). The Mare Model to Study the Effects of Ovarian Dynamics on Preantral Follicle Features. PLoS ONE.

[52] De Aguiar C.M.A., Martins S.D., Ferreira A.S., Ferreira A.S., Santos-Saboia H.E.O., Cavalcante-Filho J.E.F., Oliveira M.A.F., Moura D., Nogueira M.J., Lessa R.A. (2025). The ability of the polysaccharide extract Cissus sicyoides L. leaves to maintain normal follicle structure in ovarian tissue culture. Vitr. Cell. Dev. Biol. Anim..

[53] Livak K.J., Schmittgen T.D. (2001). Analysis of relative gene expression data using real-time quantitative PCR and the 2(-Delta Delta C(T)) Method. Methods.

